# Can the 12-item general health questionnaire be used to identify medical students who might ‘struggle’ on the medical course? A prospective study on two cohorts

**DOI:** 10.1186/1472-6920-13-48

**Published:** 2013-04-02

**Authors:** David James, Janet Yates, Eamonn Ferguson

**Affiliations:** 1 Medical Education Unit, B94 Medical School, Queen’s Medical Centre, Nottingham, NG7 2UH, UK; 2 School of Psychology, University of Nottingham, University Park, Nottingham, NG7 2RD, UK

## Abstract

**Background:**

Students who fail to thrive on the Nottingham undergraduate medical course frequently suffer from anxiety, depression or other mental health problems. These difficulties may be the cause, or the result of, academic struggling. Early detection of vulnerable students might direct pastoral care and remedial support to where it is needed. We investigated the use of the short-form General Health Questionnaire (GHQ-12) as a possible screening tool.

**Methods:**

Two consecutive cohorts (2006 and 2007) were invited to complete the GHQ-12. The questionnaire was administered online, during the second semester (after semester 1 exams) for the 2006 cohort and during the first semester for the 2007 cohort. All data were held securely and confidentially. At the end of the course, GHQ scores were examined in relation to course progress.

**Results:**

251 students entered the course in 2006 and 254 in 2007; 164 (65%) and 160 (63%), respectively, completed the GHQ-12. In both cohorts, the study and non-study groups were very similar in terms of pre-admission socio-demographic characteristics and overall course marks. In the 2006 study group, the GHQ Likert score obtained part-way through the first year was negatively correlated with exam marks during Years 1 and 2, but the average exam mark in semester 1 was the sole independent predictor of marks in semester 2 and Year 2. No correlations were found for the 2007 study group but the GHQ score was a weak positive predictor of marks in semester 2, with semester 1 average exam mark again being the strongest predictor. A post-hoc moderated-mediation analysis suggested that significant negative associations of GHQ scores with semester 1 and 2 exams applied only to those who completed the GHQ after their semester 1 exams. Students who were identified as GHQ ‘cases’ in the 2006 group were statistically less likely to complete the course on time (OR = 4.74, p 0.002). There was a non-significant trend in the same direction in the 2007 group.

**Conclusions:**

Results from two cohorts provide insufficient evidence to recommend the routine use of the GHQ-12 as a screening tool. The timing of administration could have a critical influence on the results, and the theoretical and practical implications of this finding are discussed. Low marks in semester 1 examinations seem be the best single indicator of students at risk for subsequent poor performance.

## Background

In 2006 we introduced the concept of the ‘struggling’ medical student [1]. We defined struggling in terms of academic failure, course disruption, and early course exit, alone or in combination. That and a subsequent study indicated that mental health problems, largely anxiety, depression and eating disorders, are frequently associated with struggling on the course [2], and this has been confirmed in more recent cohorts [3,4]. It is not clear whether these problems are the causes or the consequences of struggling, or quite possibly both.

The literature includes a number of studies which suggest that medical students, both in the UK and elsewhere, are potentially susceptible to stress and anxiety [5-8]. Contributory factors might include a very competitive environment, a high work-load, and patient contact. Although medical students may experience higher levels of anxiety than the general population [9], this may be common to others in a highly academic environment [10]. The majority of students will cope without difficulty, and indeed the ability to manage stress is probably essential for subsequent professional life [11]. Recent research suggests that underlying personality traits are significant in predicting both success and vulnerability to stress in medical training and beyond [12]. However, psychological screening is not routinely used in medical school admissions. In view of the associations shown between poor mental health and struggling, we speculated that early detection of over-anxious students might aid the provision of pastoral care and support. Could a simple questionnaire provide a reliable ‘flag’ for routine use?

We chose the12-item General Health Questionnaire (GHQ-12) as an investigative tool. This was developed originally as a 60-item questionnaire [13] and later reduced to a shortened form [14]. It has been used and validated extensively both in the UK and worldwide [15]. It consists of 12 questions relating to recent feelings or behaviour, focussing on mood rather than physical health. The questions are phrased in both positive and negative directions and answered by means of four-point Likert scales. The response options are worded in terms of ‘less than usual’, ‘no more than usual’, ‘rather more than usual’, or ‘much more than usual’. These are arranged so that no reverse scoring is required. The default scoring ranges from 0–3, with a maximum score of 36, and higher scores reflect increased psychiatric morbidity. As an indication of ‘caseness’, it is recommended that these scores are converted into a binary scores, such that 0 or 1 = 0, and 2 or 3 = 1, giving a maximum score of 12. A score of 4 or more has been used as a suitable cut-off point for caseness [5,8,9].

We invited two consecutive cohorts of undergraduate medical students to complete the GHQ-12 in their first year of study. The main research questions that we wished to answer were:

•What proportion of the students could be considered as ‘cases’?

•Were high GHQ scores or ‘caseness’ associated with poorer examination marks in the early course and/or with course disruption?

•Could the GHQ-12 be used as an early screening tool for those potentially at risk?

## Methods

### The study populations

The 2006 and 2007 entry cohorts were invited to complete the GHQ online. The Director of Medical Education (DJ) spoke to each cohort in advance of the study, describing its purpose, its voluntary nature, and giving reassurance that data would be fully confidential and not revealed or used outside the setting of the specific research project. Students were then sent an email with a link to full participant information (as shown in Additional file 1: Research project. General health in medical students: is there a relationship with course progress? Participant Information), which concluded with a consent button and then the on-line questionnaire. The link was active for three weeks, and during this period the students were sent a second email as a thank you for those who had completed the questionnaire and a reminder to those who had not. They were not informed of their GHQ scores after participation.

### The timing of the questionnaire

We intended to administer the questionnaire in the November of each academic year - that is about 6 weeks into the first semester. However, due to technical difficulties, the 2006 cohort was not approached until April 2007 when the students were in semester 2. However, the 2007 cohort did complete the questionnaire in semester 1. These timings were planned to avoid the main examination periods of January and late May.

### Data collection and analysis

Electronic files containing the results from the GHQ-12 were stored securely. All students making normal progress would have completed their course by June 2011 (2006 cohort) or June 2012 (2007 cohort). Separate databases were constructed for each cohort on course completion, with their available pre-admission data (socio-demographics from central university files) and course progress data (Faculty databases), together with the GHQ scores. Age at course entry was collapsed into mature (21 and over) or not (< 21); ethnicity was grouped as White vs non-White; Fee status was used as a proxy for Domicile and grouped into UK vs EU or overseas; and pre-admission qualifications were grouped into standard A-levels (including Scottish Highers) vs all others.

The course progress files of all students who had not graduated on time were inspected to determine why, and brief comments added to the database. Personal identifiers were removed from the databases on completion to provide anonymity.

Data were analysed in IBM SPSS v19 and v20. A preliminary analysis was used to determine whether the two cohorts, and the study and non-study groups within each cohort, were similar. We compared pre-admission characteristics, expressed as bivariate categories, using the *χ*^2^ test.

We also checked whether course performance was similar in the study and non-study groups of each cohort, using t-tests for average exam marks at key stages of the course (see Additional file 2 for details). The academic markers used were the average marks for Year 1, Year 2, Part I (the combined average of Years 1 and 2), Part II (the Honours course in the first half of Year 3), and separate knowledge and skills marks for each of the three parts of the clinical course (Clinical Practice (CP)1, undertaken in the second half of the third year, CP2 in the fourth year and CP3 in the fifth year).

GHQ data were examined both as the raw Likert scores and as ‘cases’. We tested the association between GHQ Likert scores and exam data, using correlation matrices (Pearson correlation coefficient). We then explored the data with hierarchical multivariate linear regression. The outcome variable was the average exam mark at either the end of semester 2 or Year 2. Explanatory variables were sex, ethnicity, domicile (as fee status), maturity, and standard A-level qualifications in block 1, the GHQ Likert score in block 2, and the semester 1 average in block 3.We also examined whether those in the study group who were ‘cases’ were more likely to have failed to complete the course on time.

#### Ethical approval

Ethical approval was obtained from the UoN Medical School Research Ethics Committee (ref A/02/2007).

## Results

### The study samples

In October 2006, 251 students commenced the course, of whom 168 (67%) completed the GHQ-12 in April 2007. However, four of these did not indicate their consent to participate in the research, and therefore the study group with GHQ data for 2006 refers to 164 students (65%) who did give their consent. In October 2007, a further 254 students commenced the course, of whom 160 (63%) gave consent and completed the GHQ-12 in November 2007.

### Comparisons of cohorts and study/non-study groups

Table 1 illustrates the pre-admission socio-demographic characteristics of the cohorts in both years. Within each cohort, pre-admission characteristics of the study and non-study groups were compared using the Chi-square test (excluding data categories marked as ‘not known’). In the 2006 groups, there were no significant differences apart from the non-study group being more likely to have non-standard A-level qualifications. The 2007 study and non-study groups showed no statistically significant differences.

**Table 1:** Pre-admission socio-demographic characteristics of the 2006 and 2007 cohorts

	**Entry cohort**		**Study group**		**Non-study group**		Chi-square test, study vs non-study groups *
	**n**	**%**	**n**	**%**	**n**	**%**	**p**
**2006 entry**	251		164		87		
Gender							
Males	94	37.5	61	37.2	33	37.9	NS
Females	157	62.5	103	62.8	54	62.1	
Age group							
Under 21	234	93.2	155	94.5	79	90.8	NS
21 or over	17	6.8	9	5.5	8	9.2	
Domicile (as Fee status)							
UK	211	84.1	137	83.5	74	85.1	NS
EU/OS	40	15.9	27	16.5	13	14.9	
Ethnicity							
White	168	66.9	116	70.7	52	59.8	NS
Non-White	80	31.9	46	28.0	34	39.1	
Not declared	3	1.2	2	1.2	1	1.1	
Last qualifications							
Standard A levels	231	92.0	157	95.7	74	85.1	0.003 †
Non-standard qualifications	20	8.0	7	5.5	13	14.9	
Not known	0	0.0	0	0.0	0	0.0	
**2007 entry**	254		160		94		
Gender							
Males	100	39.4	65	40.6	35	37.2	NS
Females	154	60.6	95	59.4	59	62.8	
Age group							
Under 21	251	98.8	158	98.8	93	98.9	NS
21 or over	3	1.2	2	1.2	1	1.1	
Domicile (as Fee status)							
UK	222	87.4	140	87.5	82	87.2	NS
EU/OS	32	12.6	20	12.5	12	12.8	
Ethnicity							
White	160	63.0	106	66.3	54	57.4	NS
Non-White	76	29.9	42	26.3	34	36.2	
Not declared	18	7.1	12	7.5	6	6.4	
Last qualifications							
Standard A levels	232	91.3	147	91.9	85	90.4	NS
Non-standard qualifications	19	7.5	12	7.5	7	7.4	
Not known	3	1.2	1	0.6	2	.2	

* for bivariate categories, excluding any unknown values.

† *X*^2^ = 8.83, Odds ratio = 3.94, 95% CI 1.51 to 10.29.

We also compared the pre-admission characteristics of the two study groups (as shown in Table 1). Apart from a smaller proportion of mature students in 2007 (χ^2^ = 4.44, OR 0.22, 95%CI 0.05 to 1.03, p = 0.035), there were no other significant differences between them.

Mean marks post-admission for the study and non-study groups were compared at each of the key stages of the course, for each cohort. Although the marks for the study groups were marginally higher than for the non-study groups in both cohorts (usually by 1–2%), these differences were not statistically significant (*t*-test). Relevant data are shown in Additional file 2: Exam performance of study and non-study groups in both cohorts.

In summary, there were no measurable differences between the study and non-study group in either cohort, apart from difference in pre-admission qualifications in the 2006 groups.

### GHQ scores

There was a very small proportion of unanswered questions; seven items (from seven students) in the 2006 cohort, and five items (from four students) in the 2007 cohort. These missing responses were scored low as recommended by the questionnaire distributors [16].

The Likert scores for both cohorts at T1 followed a near-normal distribution (K-S Z statistic = 1.34, p = 0.06 for 2006; K-S Z statistic = 1.28, p = 0.08 for 2007). For the 2006 cohort the mean Likert score was 13.39, SD 5.77, and for the 2007 cohort the mean was slightly lower, 11.48, SD 5.03. This difference was significant on t-testing (t = 3.18, p = 0.002) and suggests a lower level of anxiety overall.

**Figure 1:**
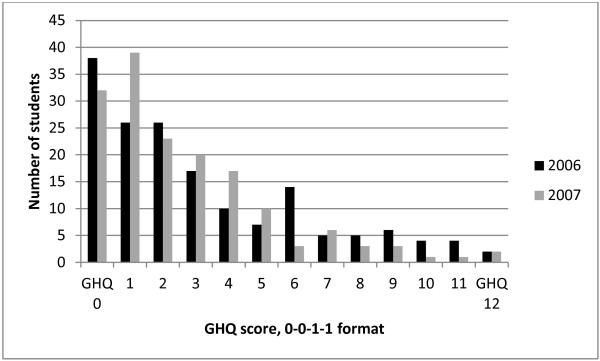
Histogram for GHQ scores (0-0-1-1 format).

The Likert scores were converted to the 0-0-1-1 format to create scores of 0–12 and hence identify ‘cases’ with a score of 4 or more. Figure 1 shows the distribution of these scores. In the 2006 cohort, 57/164 (35%) were denoted as cases, compared to 46/160 (29%) in the 2007 cohort, overall 103/324, 32%. The difference between the proportions of cases and non-cases in the two study groups was not statistically significant (*χ*^2^ test). However, the proportions with a high score of 8 or more were 21/164 (13%) in 2006 and 10/160 (6%) in 2007, which is just statistically significant (*χ*^2^ = 4.02, OR 2.20, 95% CI 1.00 to 4.83, p = 0.045).

### Correlations between GHQ scores and exam marks, and multivariate regressions

Correlations matrices between the GHQ Likert scores and early exam marks (Year 1, Year 2, Part I and Part II) for each cohort are shown in Table 2). As expected, there were strong and highly significant correlations between exam marks in year 1 and 2 (Pearson correlation coefficient r ≥ .8), and slightly lower correlations with Part II (r ~ .5 to .7). In the 2006 cohort, there was a significant negative correlation between the GHQ score and the Year 1 average (r = −.29, p < 0.001) but less with Year 2, and none with Part II. In the 2007 study group, inter-examination correlations were similar but there were no significant correlations with the GHQ.

**Table 2:** Pearson correlation coefficients for early exam marks and GHQ Likert scores

	**Year 1 average**	**Year 2 average**	**Part I**	**Part II**	**Average Parts I & II**
**2006 cohort, n = 157**					
Year 2 average	.82***				
Part I	.96***	.95***			
Part II	.54***	.69***	.64***		
Average Parts I & II	.82***	.90***	.90***	.91***	
GHQ Likert score	–.29***	–.21**	–.27**	–.08	–.19*
**2007 cohort, n = 152**					
Year 2 average	.80***				
Part I	.96***	.94***			
Part II	.52***	.59***	.58***		
Parts I & II average	.84***	.87***	.90***	.87***	
GHQ Likert score	–.02	.05	.01	.09	.05

***. Correlation is significant at the < 0.001level (2-tailed).

**. Correlation is significant at the 0.01 level (2-tailed).

*. Correlation is significant at the 0.05 level (2-tailed).

We calculated the average marks for semester 1 (S1) and semester 2 (S2) separately. This calculation excluded marks for two modules in Theme D, Early Clinical and Professional Development, and Communication Skills, which are examined largely via essays, coursework and practicals throughout the year. Average marks for the two semesters were closely correlated in the 2006 group (r = .83, p < 0.001). The GHQ correlated retrospectively with the semester 1 mark and prospectively with semester 2 (both negative, r = −.33 for S1 and -.34 for S2, both p < 0.001).

When the same analysis was conducted with the 2007 group, again there was a strong correlation between semesters 1 and 2 (r = .78, p < 0.001), but there was no significant correlation between either semester exam average and the GHQ score.

Data were examined using multivariate hierarchical linear regression, firstly with the 2006 cohort and the semester 2 average mark as the outcome variable. There were no predictors in block 1 (pre-admission characteristics). In block 2, the GHQ Likert score was a significant negative predictor (Beta – 0.34, p < 0.001), but this effect disappeared in block 3 when the semester 1 average became the sole predictor (Beta = 0.80, p < 0.001). Full data are tabulated in Additional file 3: Regression Tables for GHQ and Exam marks. When the Year 2 average was used as the outcome variable, the effects of the GHQ were still present in block 2, although less strongly, (Beta = −0.24, p = 0.004, data table in Additional file 3), but again disappeared in block 3 when the S1 mark was added (Beta for S1 mark = 0.76, p < 0.001). In summary, the GHQ did not appear to be an independent predictor of S2 or Year 2 performance once S1 results were included in the model.

For the 2007 group, multivariate linear regression for Year 1 indicated that the GHQ score was not predictive in block 2 but had a mild positive predictive effect in block 3 (Beta = 0.16, p 0.008). Again the S1 mark predominated (Beta = 0.78, p < 0.001, see Additional file 3). When the outcome variable was the Year 2 mark, the S1 mark was the sole predictor (Beta = 0.71, p < 0.001, see Additional file 3). In summary, the S1 mark was again the best predictor of S2 and Year 2 performance. The GHQ had a small positive effect in S2 only.

Socio-demographic variables did not emerge as independent predictors of performance in either year-group.

### Moderated-mediation

In view of these results, we considered the possibility that exposure to the medical school environment and examinations prior to completing the GHQ (2006 cohort) may activate the expression of GHQ-anxiety (indeed the mean Likert score is higher in this group) in a way that is not observed when the questionnaire is administered earlier (2007 cohort). This activation of anxiety may then be linked to exam performance in semester 2. We therefore carried out a *post hoc* analysis using moderated mediation Edwards and Lambert, ref [17] with the two data files combined. Full details of the technique and the resulting statistics are presented in Additional file 4. In essence this confirmed that the negative correlation between GHQ and semester 1 exam and semester 2 exam results respectively was only observed when the GHQ was administered after the semester 1 exams.

### GHQ ‘caseness’ and course completion

We flagged all students on the databases as successful (completed the course on time, or that any delay was due to legitimate causes such as completion of further research after the BMedSci project), or less successful (left prematurely or suffered performance-related course disruption). In 2006, 147/164 (90%) of those in the study group completed successfully, compared to 76/87 (87%) of the non-study group (*χ*^2^ = NS). In 2007 the comparable figures were 147/160 (92%) and 84/94 (89%) (*χ*^2^ = NS).

We then compared the proportion of GHQ cases, scored either at ≥ 4 or ≥ 8, who were less successful (Table 3). In 2006, significantly more cases than non-cases failed to complete the course at both thresholds. In 2007, only a score of 8 was significantly more common in the less successful group though the trend was in the same direction as for a score of ≥ 4.

**Table 3:** GHQ ‘caseness’ and course completion

**Entry Year**		**Non-case (score < 4)**		**Case (score ≥ 4)**		***χ***^**2**^	**OR**	**95% CI**	**p**
		**n**	**%**	**n**	**%**				
2006	Successful student *	101	94.4	46	80.7	7.50	4.03	1.40–11.55	0.006
	Less successful student †	6	5.6	11	19.3				
2007	Successful student *	108	94.7	40	87.0				NS
	Less successful student †	6	5.3	6	13.0				
		**Non-case (score < 8)**		**Case (score ≥ 8)**					
2006	Successful student *	133	93.0	14	66.7				0.002 ‡
	Less successful student †	10	7.0	7	33.3				
2007	Successful student *	141	94.0	7	70.0				0.03 ‡
	Less successful student †	9	6.0	3	30.0				

* student completed on time, or legitimate reason for delay in high-performing student.

† student failed to complete on time due to course disruption or attrition.

‡ Fisher’s exact test.

## Discussion

This two-cohort pilot study revealed interesting and useful results. In the 2006 group, tested mid-way between Semester 1 and 2 exams, higher scores on the GHQ were significantly correlated with lower overall marks in years 1 and 2 on univariate analysis. In the 2007 group, tested early in the course and before any exams had been taken, the GHQ scores tended to be slightly lower and no such univariate correlations were found. With multivariate analysis, the GHQ did not predict S2 results in the 2006 cohort once S1 results were included, and in 2007 it only had a small independent predictive effect on S2 results. In both years the S1 results were the best independent predictors of S2 results. ‘Caseness’ at ≥ 4 or ≥ was significantly associated with course disruption or failure in the 2006 cohort, but this was only at the ≥ 8 threshold in the 2007 group.

The overall caseness rate of 32% for the two cohorts seems high. However, it is comparable with reports from other medical schools. Direct comparison with other studies is affected by variations in the student cohorts, the timing and type of questionnaire, response rates, and study objectives. An early study in one UK medical school gave a figure of 37% caseness in the first year (84% response rate), but correlations with performance were not provided [5]. A comparable study in a Scottish school using a problem-based learning (PBL) curriculum found 25% caseness (70% response rate) in the first term but again without data on correlation with course performance [8]. A study at another Scottish school found 28% caseness towards the end of the 1st year and a small negative association between GHQ scores and exam ranking [18]. However, as that survey covered all five academic year-groups, and had a low response rate of only 40%, interpretation is difficult.

The question remains as to why the relationships between the GHQ scores and early course performance differed in the two cohorts. The pre-admission characteristics that we measured were broadly similar and it is unlikely that the few differences observed account for contrasting outcomes. One inherent change was that the admissions procedure in 2007 included scores from the UKCAT (United Kingdom Admissions Test). As this is a test of general intellectual ability, and has been shown not to influence national admissions very markedly [19], nor to be predictive of early course performance at Nottingham [20], it is unlikely to account for our current data. However, we cannot exclude the possibility that the two cohorts differed in some way that we did not measure.

The other possible explanation lies in the timing of the questionnaire administration. The 2006 cohort had completed the semester 1 exams in January and the students would be taking their semester 2 exams in 4–6 weeks. It is quite possible that some were experiencing exam-related stress, especially those who had performed less well in January. The 2007 cohort, on the other hand, had been on the course for only 6–8 weeks, well before the first exams. Those who were GHQ ‘cases’ may have been affected by anxiety secondary to settling in to University life, living away from home and having to make new friends, but probably unrelated to exams. The wording of the GHQ may also be a factor in the interpretation of these results. It asks questions in terms of comparison with ‘usual’ feelings, so may be sensitive to stressful events such as imminent examinations. Indeed, there is some evidence that context can cue responding to questionnaires [21,22].

Current thinking on personality theory within the context of behavioural reaction norms [23,24] and socio-genomics [25] suggests that environmental factors may serve to activate trait based expression (see also [26,27]). As such, the exposure to exam stress before completing the GHQ may have served to activate the expression of this trait in the 2006 cohort, in a way that had not yet occurred in the 2007 cohort who completed the GHQ prior to exams. Those in the 2006 cohort had significantly higher scores on the Likert GHQ than those in the 2007 cohort and this activation have influenced subsequent exam scores. While the analyses are consistent with this interpretation it needs to be acknowledged that students were not randomly allocated to GHQ administration time and the effect may represent a cohort effect. As we cannot exclude the possibility that the two cohorts differed in some way that we did not measure we cannot make any causal claims concerning the timing of the administration of the GHQ relative to the exams taken on the relationships observed. However, the results suggest that there may be appropriate sensitive periods to assess traits such as anxiety to improve their prognostic value.

The small but significant positive relationship between the GHQ and S2 marks in the 2007 cohort may reflect the “approach” nature of anxiety, which may help people to move towards and explore threatening contexts, in order to identify ways to deal with them [28,29].

### Limitations of the study

This study concerns two year groups at one medical school so would not necessarily be generalizable to other schools with different intakes and curricula. In addition, only 63–65% of the intakes responded, so an underlying response bias could have affected results. However, there were no major differences in measurable criteria between cohorts or study groups, and our data for the percentage of students being classified as ‘cases’ was similar to that obtained elsewhere.

As mentioned in the Discussion above, the GHQ tends to identify ‘state anxiety’ (by asking how respondents have been feeling over the last few weeks) rather than ‘trait anxiety’ (how they feel most of the time). It is therefore unsurprising that the results could be dependent on the exact timing of administration. Repeated administration of the questionnaire at different times during the academic year would be required to clarify this aspect.

## Conclusions

The results from two cohorts provide insufficient evidence to recommend the routine use of the GHQ-12 as a screening tool. The timing of administration may have a critical influence on the results, and the theoretical and practical implications of this finding are discussed. Low marks in semester 1 examinations appear to be the best indicator of students at risk for subsequent poor performance.

## Competing interests

The authors declare that they have no competing interests.

## Authors’ contributions

All authors contributed to devising the study, interpreting the data, and writing the paper. JY collected and analysed the data and wrote the first draft of the paper and EF performed the path modelling. All authors approved the final version.

## Pre-publication history

The pre-publication history for this paper can be accessed here:

http://www.biomedcentral.com/1472-6920/13/48/prepub

## Supplementary Material

Additional file 1 **Research project.** General health in medical students: is there a relationship with course progress? Participant Information. Participant information as approved by the Ethics Committee and provided to the students. Click here for file

Additional file 2 **Exam performance of study and non-study groups in both cohorts.** This Table shows the average exam data (mean and SD) throughout the course for the study and non-study groups in both cohorts. Click here for file

Additional file 3 **Regression Tables for GHQ and Exam marks.** Tables 3.1 and 3.2 show the multivariate hierarchical regression data for both cohorts, using semester 2 exam data as the dependent variable. Tables 3.3 and 3.4 show the equivalent data when the Year 2 average is the outcome variable. Click here for file

Additional file 4 **Moderated-mediation Analysis.** This file explains the statistical modelling technique of moderated-mediation as applied to the data. It provides tabulated coefficients (Table 4.1), and a visual model of the effects seen (Figure 4.1), together with relevant references. Click here for file
