# Supplementary material for: Can the 12-item general health questionnaire be used to identify medical students who might ‘struggle’ on the medical course? A prospective study on two cohorts
Source: BMC Med Educ. 2013 Apr 2;13:48. doi: 10.1186/1472-6920-13-48 (PMC3616988; doi:10.1186/1472-6920-13-48)
Supplement: Additional file 1 — Research project. General health in medical students: is there a relationship with course progress? Participant Information. Participant information as approved by the Ethics Committee and provided to the students. [file 1472-6920-13-48-S1.doc]

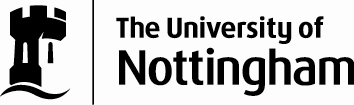


**Faculty of Medicine and Health Sciences**

**Research project**

**General health in medical students: is there a relationship with course progress?**

**Participant Information**

Dear Student

I would like to invite you to participate in our ongoing research programme in the Medical Education Unit.

We know that a number of factors can influence students’ progress during their course, including their academic ability, personality, and learning styles. Whilst the majority of students enjoy a happy and successful time on the course, there are a few each year who will suffer difficulties, sometimes associated with health problems.

I would like to explore this relationship more closely and am asking for YOUR help in our research. I would be grateful if you would complete this very brief on-line General Health questionnaire – it will only take a couple of minutes.

The results will be linked anonymously to examination results and used in aggregate form for research purposes ONLY. The data will be held securely and confidentially on a password-protected anonymised research database, and will NOT be accessible to anyone in the Faculty nor disclosed to academic staff or tutors. The overall outcome of the research may assist in the development of pastoral support for medical students in the future.

**Approval for this Questionnaire has been given by the Medical School Research Ethics Committee.**

**I would like to emphasise these points:**

- You answers will be held securely and confidentially on a password-protected database on the University file servers, and analysed in the Medical Education Unit for research purposes only. The data will NOT be used for your appraisal or pastoral support in the course and no member of the Faculty staff will know whether or not you have participated.
- The data will be linked electronically to examination scores and general progress on the course, but this information will be used in aggregate form for research and development purposes ONLY and individuals will not be identified. Your name will not be held on the database, only your University ID number and a unique study ID.
- As the Research Fellow in the MEU, I have no involvement whatsoever with the teaching or assessment of the course, nor any personal knowledge of students. It will not be possible for any members of Faculty staff to access the database or to obtain information about individuals.
- Your participation is entirely voluntary and can withdraw at any point even if you have agreed initially; you can ask for your data to be withdrawn later
- Your participation (or not) will not in any way influence your progress or assessment on the course
- If you are a BMedSci student, the information that you may have given in the Learning Styles Questionnaire last summer may also be linked to the anonymised database and used in aggregated analysis.

If you have any questions or require further information about this research, you may contact me as shown below.

If you are happy to continue, then please check the box below to indicate your consent. Remember, agreeing now does not mean you have to complete the study.

THANK YOU for helping with this important research – your participation is important to us.

Janet Yates

Research Fellow

Medical Education Unit

B94, QMC

Email: janet.yates@nottingham.ac.uk

Tel: 0115 8230029 (University extension 30029)

Mon-Weds only, 8am-4pm

……………………………………………………………………………………………………………………………………………

**Consent statement:**

*“I confirm that I have read this Participant Information, and that I understand the nature and purpose of this Questionnaire which I agree to complete.”* [electronic check box]

…………………………………………………………………………………………………………………………………………………

**Further help and advice:**

Should you have any concerns about your health after answering these questions, please seek advice or assistance from the appropriate source. There is a comprehensive list in your pastoral care booklet, but we include a few key numbers below:

For illness, contact your own GP initially

| **University services** | **Outside Agencies** |
| --- | --- |
| Your Personal Tutor | Alcohol problems Advisory Service  0115 941 4747 |
| University Counselling Service  0115 951 3695 | Citizens Advice Bureau  0115 958 9330 |
| Nightline (7pm – 8 am)  0115 95 14985 | Disablement Information Advice Lines  0115 950 5656 |
| University Chaplains  0115 95 13930 | GU Medicine, City Hospital  0115 962 7747 (women) or 962 7745 (men) |
| Occupational Health Service  0115 951 4329 | Health Shop  0115 947 5414 |
| Cripps Health Centre  0115 846 8888 | Rape Crisis Centre  0115 941 0440 |
| Student Advice Centre  0115 935 1142 | Samaritans  08457 909090 |
