# Supplementary material for: Can the 12-item general health questionnaire be used to identify medical students who might ‘struggle’ on the medical course? A prospective study on two cohorts
Source: BMC Med Educ. 2013 Apr 2;13:48. doi: 10.1186/1472-6920-13-48 (PMC3616988; doi:10.1186/1472-6920-13-48)
Supplement: Additional file 2 — Exam performance of study and non-study groups in both cohorts. This Table shows the average exam data (mean and SD) throughout the course for the study and non-study groups in both cohorts. [file 1472-6920-13-48-S2.docx]

**Additional File 2: Exam performance of study and non-study groups in both cohorts**

|  |  | **2006 cohort** | | | **2007 cohort** | | |
| --- | --- | --- | --- | --- | --- | --- | --- |
|  | **Student group** | **n** | **Mean** | **Std. Deviation** | **n** | **Mean** | **Std. Deviation** |
| Year 1 average | Study | 157 | 63.6 | 8.3 | 152 | 64.6 | 8.1 |
|  | Non-study | 83 | 63.0 | 8.8 | 87 | 63.9 | 8.2 |
| Year 2 average | Study | 157 | 65.1 | 7.0 | 152 | 65.5 | 7.0 |
|  | Non-study | 83 | 64.7 | 7.5 | 87 | 64.6 | 7.5 |
| Part I (average Year 1 + Year 2) | Study | 157 | 64.4 | 7.3 | 152 | 65.1 | 7.2 |
|  | Non-study | 83 | 63.9 | 7.8 | 87 | 64.3 | 7.4 |
| Part II (Honours course) | Study | 157 | 65.2 | 5.5 | 152 | 52.7 | 4.2 |
|  | Non-study | 83 | 64.7 | 5.2 | 87 | 52.7 | 4.2 |
| Average Parts I & II | Study | 157 | 64.9 | 5.7 | 152 | 57.7 | 4.8 |
|  | Non-study | 83 | 64.3 | 5.8 | 87 | 57.3 | 4.9 |
| CP1 knowledge | Study | 160 | 64.4 | 10.2 | 152 | 62.2 | 9.5 |
|  | Non-study | 85 | 62.1 | 8.6 | 87 | 61.0 | 9.3 |
| CP1 skills | Study | 159 | 73.4 | 14.3 | 152 | 73.9 | 12.9 |
|  | Non-study | 85 | 70.5 | 14.2 | 87 | 72.9 | 12.6 |
| CP2 knowledge | Study | 156 | 63.4 | 7.4 | 142 | 65.0 | 6.1 |
|  | Non-study | 84 | 62.9 | 7.9 | 85 | 65.5 | 6.5 |
| CP2 skills | Study | 156 | 64.4 | 6.6 | 142 | 65.7 | 6.2 |
|  | Non-study | 84 | 63.9 | 6.6 | 85 | 65.6 | 6.5 |
| CP3 knowledge | Study | 141 | 66.8 | 6.4 | 142 | 65.8 | 5.9 |
|  | Non-study | 76 | 66.3 | 6.0 | 84 | 66.0 | 4.9 |
| CP3 skills | Study | 141 | 68.0 | 5.7 | 142 | 67.6 | 4.9 |
|  | Non-study | 76 | 67.3 | 5.2 | 84 | 68.1 | 4.9 |
