# Supplementary material for: Can the 12-item general health questionnaire be used to identify medical students who might ‘struggle’ on the medical course? A prospective study on two cohorts
Source: BMC Med Educ. 2013 Apr 2;13:48. doi: 10.1186/1472-6920-13-48 (PMC3616988; doi:10.1186/1472-6920-13-48)
Supplement: Additional file 3 — Regression Tables for GHQ and Exam marks. Tables 3.1 and 3.2 show the multivariate hierarchical regression data for both cohorts, using semester 2 exam data as the dependent variable. Tables 3.3 and 3.4 show the equivalent data when the Year 2 average is the outcome variable. [file 1472-6920-13-48-S3.docx]

Additional File 3: Regression Tables for GHQ and Exam marks

**Table 3.1: Multivariate hierarchical linear regression, 2006 cohort.**

**Outcome variable: Semester 2 exams**

|  |  | **Unstandardized Coefficients** | | **Standardized Coefficients** | |  | **95.0% Confidence Interval for B** | |
| --- | --- | --- | --- | --- | --- | --- | --- | --- |
| **Block** |  | **B** | **Std. Error** | **Beta** | **t** | **Sig.** | **Lower Bound** | **Upper Bound** |
|  |  |  |  |  |  |  |  |  |
| 1 | Mature, 21 or over | 0.38 | 4.50 | 0.01 | 0.08 | 0.934 | -8.51 | 9.26 |
|  | Male sex | 0.24 | 1.89 | 0.01 | 0.13 | 0.898 | -3.48 | 3.97 |
|  | Home student | -3.93 | 2.69 | -0.13 | -1.46 | 0.146 | -9.23 | 1.38 |
|  | White ethnicity | 2.90 | 2.21 | 0.12 | 1.31 | 0.191 | -1.46 | 7.25 |
|  | Standard A-level or Highers | 4.83 | 5.13 | 0.09 | 0.94 | 0.347 | -5.29 | 14.96 |
|  |  |  |  |  |  |  |  |  |
| 2 | Mature, 21 or over | -1.33 | 4.28 | -0.03 | -0.31 | 0.757 | -9.78 | 7.13 |
|  | Male sex | -1.42 | 1.83 | -0.06 | -0.78 | 0.439 | -5.02 | 2.19 |
|  | Home student | -2.54 | 2.57 | -0.08 | -0.99 | 0.324 | -7.61 | 2.53 |
|  | White ethnicity | 0.74 | 2.15 | 0.03 | 0.35 | 0.730 | -3.50 | 4.98 |
|  | Standard A-level or Highers | 4.93 | 4.86 | 0.09 | 1.01 | 0.312 | -4.67 | 14.52 |
|  | GHQ Likert score | -0.68 | 0.16 | **-0.34** | **-4.35** | **<0.001** | -0.99 | -0.37 |
|  |  |  |  |  |  |  |  |  |
| 3 | Mature, 21 or over | -2.21 | 2.59 | -0.04 | -0.85 | 0.396 | -7.33 | 2.91 |
|  | Male sex | 0.71 | 1.11 | 0.03 | 0.64 | 0.525 | -1.49 | 2.91 |
|  | Home student | 0.37 | 1.56 | 0.01 | 0.24 | 0.814 | -2.72 | 3.46 |
|  | White ethnicity | -0.29 | 1.30 | -0.01 | -0.22 | 0.825 | -2.86 | 2.28 |
|  | Standard A-level or Highers | -1.34 | 2.96 | -0.02 | -0.45 | 0.652 | -7.20 | 4.52 |
|  | GHQ Likert score | -0.14 | 0.10 | -0.07 | -1.38 | 0.169 | -0.34 | 0.06 |
|  | Average of S1 exams | 0.98 | 0.06 | **0.80** | **16.40** | **<0.001** | 0.86 | 1.09 |
| a. Dependent Variable: Average of S2 exams | | |  |  |  |  |  |  |

**Table 3.2: Multivariate hierarchical linear regression, 2007 cohort.**

**Outcome variable: Semester 2 exams**

|  |  | **Unstandardized Coefficients** | | **Standardized Coefficients** | | | **95.0% Confidence Interval for B** | |
| --- | --- | --- | --- | --- | --- | --- | --- | --- |
| **Block** |  | **B** | **Std. Error** | **Beta** | **t** | **Sig.** | **Lower Bound** | **Upper Bound** |
|  |  |  |  |  |  |  |  |  |
| 1 | Male sex | 1.74 | 1.75 | 0.08 | 0.99 | 0.322 | -1.72 | 5.20 |
|  | Mature, 21 or over | -2.53 | 10.98 | -0.02 | -0.23 | 0.818 | -24.23 | 19.18 |
|  | Home student | -0.79 | 3.32 | -0.03 | -0.24 | 0.813 | -7.35 | 5.78 |
|  | White ethnicity | -2.36 | 2.10 | -0.11 | -1.12 | 0.264 | -6.51 | 1.79 |
|  | Standard A-level or Highers | 3.08 | 4.09 | 0.08 | 0.75 | 0.453 | -5.01 | 11.17 |
|  |  |  |  |  |  |  |  |  |
| 2 | Sex | 1.85 | 1.81 | 0.09 | 1.02 | 0.309 | -1.73 | 5.43 |
|  | Mature, 21 or over | -2.18 | 11.10 | -0.02 | -0.20 | 0.845 | -24.13 | 19.77 |
|  | Home student | -0.83 | 3.34 | -0.03 | -0.25 | 0.805 | -7.42 | 5.77 |
|  | White ethnicity | -2.31 | 2.11 | -0.10 | -1.09 | 0.276 | -6.49 | 1.87 |
|  | Standard A-level or Highers | 3.20 | 4.13 | 0.08 | 0.77 | 0.440 | -4.97 | 11.37 |
|  | GHQ Likert score | 0.05 | 0.19 | 0.02 | 0.25 | 0.805 | -0.34 | 0.43 |
|  |  |  |  |  |  |  |  |  |
| 3 | Male sex | -0.42 | 1.18 | -0.02 | -0.35 | 0.724 | -2.76 | 1.92 |
|  | Mature, 21 or over | -0.20 | 7.19 | 0.00 | -0.03 | 0.978 | -14.42 | 14.02 |
|  | Home student | -0.04 | 2.16 | 0.00 | -0.02 | 0.985 | -4.31 | 4.23 |
|  | White ethnicity | -0.46 | 1.38 | -0.02 | -0.33 | 0.739 | -3.18 | 2.26 |
|  | Standard A-level or Highers | 0.83 | 2.68 | 0.02 | 0.31 | 0.758 | -4.47 | 6.13 |
|  | GHQ Likert score | 0.34 | 0.13 | **0.16** | **2.70** | **0.008** | 0.09 | 0.60 |
|  | Average mark for S1 * | 0.85 | 0.06 | **0.78** | **13.91** | **<0.001** | 0.73 | 0.97 |
| a. Dependent Variable: Average mark for S2 * | | | | | | | | |

* S1 = Semester 1 exams; S2 = Semester 2 exams

**Table 3.3: Multivariate hierarchical linear regression, 2006 cohort**

**Outcome variable: Year 2 exams**

|  |  |  |  | **Coefficients(a)** | |  |  |  |
| --- | --- | --- | --- | --- | --- | --- | --- | --- |
|  |  | **Unstandardized Coefficients** | | **Standardized Coefficients** | |  | **95.0% Confidence Interval for B** | |
| **Block** |  | **B** | **Std. Error** | **Beta** | **t** | **Sig.** | **Lower Bound** | **Upper Bound** |
|  |  |  |  |  |  |  |  |  |
| 1 | Mature, 21 or over | 1.58 | 2.98 | 0.05 | 0.53 | 0.598 | -4.32 | 7.47 |
|  | Male sex | -2.36 | 1.14 | -0.17 | -2.08 | 0.040 | -4.61 | -0.11 |
|  | Home student | -0.33 | 1.64 | -0.02 | -0.20 | 0.841 | -3.57 | 2.91 |
|  | White ethnicity | 3.04 | 1.38 | 0.20 | 2.21 | 0.029 | 0.32 | 5.76 |
|  | Standard A-level or Highers | 2.68 | 3.48 | 0.08 | 0.77 | 0.441 | -4.19 | 9.55 |
|  |  |  |  |  |  |  |  |  |
| 2 | Mature, 21 or over | 0.71 | 2.93 | 0.02 | 0.24 | 0.808 | -5.07 | 6.49 |
|  | Male sex | -3.11 | 1.14 | -0.22 | -2.73 | 0.007 | -5.36 | -0.86 |
|  | Home student | -0.10 | 1.60 | -0.01 | -0.07 | 0.949 | -3.27 | 3.06 |
|  | White ethnicity | 2.50 | 1.36 | 0.16 | 1.84 | 0.067 | -0.18 | 5.18 |
|  | Standard A-level or Highers | 3.11 | 3.40 | 0.09 | 0.92 | 0.361 | -3.60 | 9.82 |
|  | GHQ Likert score | -0.30 | 0.10 | **-0.24** | **-2.91** | **0.004** | -0.50 | -0.10 |
|  |  |  |  |  |  |  |  |  |
| 3 | Mature, 21 or over | -2.12 | 1.94 | -0.07 | -1.10 | 0.275 | -5.95 | 1.70 |
|  | Male sex | -1.81 | 0.76 | -0.13 | -2.40 | 0.018 | -3.31 | -0.32 |
|  | Home student | 1.65 | 1.06 | 0.09 | 1.55 | 0.122 | -0.45 | 3.75 |
|  | White ethnicity | 1.56 | 0.90 | 0.10 | 1.74 | 0.084 | -0.21 | 3.33 |
|  | Standard A-level or Highers | -1.20 | 2.26 | -0.03 | -0.53 | 0.597 | -5.66 | 3.26 |
|  | GHQ Likert score | -0.01 | 0.07 | -0.01 | -0.08 | 0.936 | -0.15 | 0.13 |
|  | Average of S1 exams | 0.59 | 0.04 | **0.76** | **13.96** | **<0.001** | 0.51 | 0.67 |
| a. Dependent Variable: Year 2 average | | | | | | | | |

**Table 3.4: Multivariate hierarchical linear regression, 2007 cohort**

**Outcome variable: Year 2 exams**

|  |  | **Unstandardized Coefficients** | | **Standardized Coefficients** | | | **95.0% Confidence Interval for B** | |
| --- | --- | --- | --- | --- | --- | --- | --- | --- |
| **Block** |  | **B** | **Std. Error** | **Beta** | **t** | **Sig.** | **Lower Bound** | **Upper Bound** |
|  |  |  |  |  |  |  |  |  |
| 1 | Male sex | -0.06 | 1.20 | 0.00 | -0.05 | 0.961 | -2.44 | 2.32 |
|  | Mature, 21 or over | -4.85 | 7.40 | -0.06 | -0.66 | 0.513 | -19.48 | 9.78 |
|  | Home student | -0.48 | 2.25 | -0.02 | -0.21 | 0.832 | -4.92 | 3.96 |
|  | White ethnicity | -0.65 | 1.44 | -0.04 | -0.45 | 0.653 | -3.49 | 2.20 |
|  | Standard A-level or Highers | 2.46 | 2.76 | 0.09 | 0.89 | 0.374 | -2.99 | 7.91 |
|  |  |  |  |  |  |  |  |  |
| 2 | Male sex | -0.03 | 1.24 | 0.00 | -0.03 | 0.980 | -2.48 | 2.42 |
|  | Mature, 21 or over | -4.75 | 7.49 | -0.06 | -0.64 | 0.527 | -19.56 | 10.05 |
|  | Home student | -0.49 | 2.26 | -0.02 | -0.22 | 0.828 | -4.96 | 3.98 |
|  | White ethnicity | -0.63 | 1.45 | -0.04 | -0.44 | 0.664 | -3.51 | 2.24 |
|  | Standard A-level or Highers | 2.49 | 2.79 | 0.09 | 0.89 | 0.373 | -3.02 | 8.01 |
|  | GHQ Likert score | 0.01 | 0.14 | 0.01 | 0.10 | 0.923 | -0.26 | 0.28 |
|  |  |  |  |  |  |  |  |  |
| 3 | Male sex | -1.78 | 0.92 | -0.13 | -1.93 | 0.055 | -3.60 | 0.04 |
|  | Mature, 21 or over | -3.82 | 5.47 | -0.05 | -0.70 | 0.486 | -14.64 | 7.00 |
|  | Home student | -0.02 | 1.65 | 0.00 | -0.01 | 0.993 | -3.28 | 3.25 |
|  | White ethnicity | 0.31 | 1.07 | 0.02 | 0.30 | 0.769 | -1.79 | 2.42 |
|  | Standard A-level or Highers | 0.80 | 2.04 | 0.03 | 0.39 | 0.695 | -3.24 | 4.85 |
|  | GHQ Likert score | 0.15 | 0.10 | 0.10 | 1.45 | 0.149 | -0.05 | 0.34 |
|  | Average for S1 | 0.53 | 0.05 | **0.71** | **10.90** | **<0.001** | 0.43 | 0.62 |
| a. Dependent Variable: Year 2 Average | | | | | | | | |
