# Supplementary material for: Can the 12-item general health questionnaire be used to identify medical students who might ‘struggle’ on the medical course? A prospective study on two cohorts
Source: BMC Med Educ. 2013 Apr 2;13:48. doi: 10.1186/1472-6920-13-48 (PMC3616988; doi:10.1186/1472-6920-13-48)
Supplement: Additional file 4 — Moderated-mediation Analysis. This file explains the statistical modelling technique of moderated-mediation as applied to the data. It provides tabulated coefficients (Table 4.1), and a visual model of the effects seen (Figure 4.1), together with relevant references. [file 1472-6920-13-48-S4.docx]

**Additional file 4: Moderated-mediation analysis**

To examine the relationships between the GHQ, administration time and exam marks more formally we ran a *post hoc* 1^st^ and 2^nd^ stage moderated-mediation model. This explores whether the pattern of results observed in the separate regressions hold in a single model [1], using the two datasets together.

Mediation addresses a particular type of ‘causal’ model with two causal paths. The first – the direct path - examines the direct link between the predictor (X) and the outcome (Y) and the second – the indirect path – specifies a path where X is linked to Y via a third variable known as a mediator (M), whereby X causes M and M causes Y. If the relationship between X and Y disappears when M is controlled then the X-Y relationship is fully mediated by M [2, 3].

Moderation also explores the effect of the third variable on the X-Y relations and asks if the relationship between X and Y varies as a function of a third moderator variable (Z). For example, the relationship between stress (X) and health (Y) is stronger for those who have higher levels of anxiety (Z) compared to those with lower levels. These two analytic procedures can be combined into moderated-mediation and mediated-moderation models [1, 4, 5]. The former asks if the links in the mediation models (X-M, M-Y and X-Y) are themselves open to moderation (e.g., differ for different sub-groups, levels of a trait etc.). The later asks if a moderation effect (ZX-Y) is mediated via M. In these analyses we are concerned with a moderated-mediation and in particular a 1^st^ and 2^nd^ stage model examining if the X-M and M-Y links are moderated by the timing of the administration of the GHQ relative to sitting exams. The moderated-mediation model examined whether the link between semester 1 (X) and semester 2 marks (Y) was mediated by GHQ anxiety (M), and also whether the links between semester 1 exams and GHQ-anxiety (X-M), and GHQ-anxiety and semester 2 exams (M-Y), were moderated by the timing of the administration of the GHQ (‘early’ in the 2007 cohort before semester 1 exams and ‘late’ in the 2006 cohort, after semester 1 exams) (Z). As administration time was not randomly allocated but confounded with cohort it was necessary to show first that the 2006 and 2007 cohorts did not differ on demographics and exam performance. Analyses showed no significant differences (all p > 0.17), except for slightly more students over 21 in the 2006 cohort (χ^2^ (1) = 4.44, OR 0.218, 95%CI 0.046 to 1.025, p = .035). Holding maturity as a covariate did not significantly alter the moderation mediation results.

Hayes’ (2012) PROCESS routine (<http://www.afhayes.com/spss-sas-and-mplus-macros-and-code.html>) was used to implement the 1^st^ and 2^nd^ stage moderated-mediation model which is shown in Table 4.1 and represented diagrammatically in Figure 4.1. Examining the unstandardized coefficients the results show that semester 1 exam results significantly predicted semester 2 results (B = 0.91 p < .001), that there was a significant negative association between semester 1 exam results and GHQ anxiety (B = -0.13 p < .001), indicating that the relationship between semester 1 exams and GHQ is weaker when the GHQ is administered in semester 1 rather than after the exams in semester 2. GHQ anxiety was not significantly associated with semester 2 exam results (B = 0.07 ns).

However, there were significant 1^st^ stage (semester 1 with GHQ exams: B = 0.13, *p* < .05) and 2^nd^ stage (GHQ with semester 2 exams: B = 0.50, p < .01) moderation effects depending on the time of administration of the GHQ. In other words, the 1^st^ stage moderation effect showed that the negative link between semester 1 exams results and GHQ was significantly different depending on when the GHQ was administered.

The nature of this 1^st^ stage interaction was examined by exploring the association between semester 1 exam results and GHQ anxiety separately in additional regression analyses. The results showed a significant negative association between semester 1 exams and GHQ anxiety in those who completed the GHQ later in semester 2 after the semester 1 exams (*β* = -.33, *p* < .001): That is, in this group those who did better in their semester 1 exams had lower GHQ scores in semester 2. In contrast, this association was non-significant for those who completed the GHQ earlier in semester 1 (*β* = -.14).

While the link between GHQ and semester 2 exams was non-significant, it was moderated by time of administration. The nature of the 2^nd^ stage moderation was examined in the same way as the first stage moderation. For those who completed the GHQ later (in semester 2 after semester 1 exams) the association between GHQ anxiety and exam performance in semester 2 was significant (*β* = -.34, *p* <.001). That is for this group higher GHQ scores were linked to lower exam performance. This was not the case for those who completed the GHQ earlier (*β* = .02) in semester 1. Thus while semester 1 exams predict semester 2 exams, there is an indirect effect via GHQ scores only if the GHQ is assessed after the initial exams, suggesting that initial exam stress may activate anxiety which has an influence on subsequent exams marks.

**Table 4.1: Moderated-Mediation Analyses**

|  | **GHQ (M)** | **Semester 2 exams (Y)** |
| --- | --- | --- |
|  |  |  |
| GHQ (M) |  | 0.07 |
| Semester 1 exams (X) | -0.13*** | 0.91*** |
| Administration time (Z) | -2.01** | 1.17 |
| Interaction (XZ) | 0.13* | 0.50** |
| GHQ (M) |  | 0.07 |
| R2 | .11*** | .66*** |

*Note*. Administration Time refers to which semester the GHQ was administered where 0 = semester 2 after semester 1 exams (the 2006 cohort) and 1 = semester 1 prior to semester 1 exams (the 2007 cohort).

Coefficients are unstandardised. * p < 0.05, ** p < 0.01, *** p < 0.001

References

1. Edwards J, Lambert L: **Methods for integrating moderation and mediation: a general analytical framework using moderated path analysis**. *Psychological Methods* 2007, **12**:1-22.

2. MacKinnon D, Fairchild A, Fritz M: **Mediation analysis**. *Ann Rev Psychol* 2007, **58**:593-614.

3. MacKinnon D, Krull J, Lockwood C: **Equivalence of the mediation, confounding and suppression effect**. *Prev Sci* 2000, **1**:173-181.

4. Fairchild A, MacKinnon D: **A general model for testing mediation and moderation effects**. *Prev Sci* 2009, **10**:87-99.

5. Muller D, Judd C, Yzerbyt V: **When moderation is mediated and mediation is moderated**. *J Pers Soc Psychol* 2005, **89**:852-863.

**Figure 4.1: Moderated-Mediation Model of interaction between administration of the GHQ and Semester exams**

Administration Time

Administration Time

GHQ-Anxiety

0.13*

0.07

0.50**

-0.13***

0.91***

Semester 2 Exams

Semester 1 Exams

*Note*. Administration Time refers to which semester the GHQ was administered where 0 = semester 2 (the 2006 cohort) and 1 = semester 1 (the 2007 cohort).

Coefficients are unstandardised. * = p < 0.05, ** = p < 0.01, *** = p < 0.001
